# A new method to evaluate floodwater for control/use in high-sediment rivers of Northwest China

**DOI:** 10.1038/s41598-017-17489-6

**Published:** 2017-12-08

**Authors:** Xungui Li

**Affiliations:** 1 Key Laboratory of Western China’s Environmental Systems (Ministry of Education), College of Earth and Environmental Sciences, Lanzhou University, 222 South Tianshui Road, Lanzhou, Gansu Province 730000 China; 2 Research Center for Arid Region and Desert, Lanzhou University, 222 South Tianshui Road, Lanzhou, Gansu Province 730000 China

## Abstract

Evaluating the quantity of flood season floodwater that is difficult to control or use in rivers with high sediment concentration is an important part of water resource evaluation and floodwater resource utilisation. This study proposes a method coupling water quantity and quality to evaluate such floodwater. The method divides floodwater into floodwater that is difficult to control (‘difficult-controllable’) and floodwater that is controllable but difficult to use (‘controllable but difficult-use’). A case study of the Bajiazui Reservoir in the Jing River in China’s Loess Plateau is presented. The average annual quantity of difficult-controllable floodwater is 10.4 million m^3^. The annual mean quantity of the difficult-controllable/-use floodwater is 38.1 million m^3^. The majority of that amount (78.21%) comprised controllable but difficult-use floodwater. An analysis of 64 combinations of factors influencing the quantity of the difficult-controllable/-use floodwater shows that the sediment concentration of run-off is the primary factor influencing the difficult-controllable/-use floodwater. The reservoir’s maximum flood release capacity and floodwater rejection coefficient are the primary factors affecting the difficult-controllable and controllable but difficult-use floodwater, respectively. The new evaluation method is superior to traditional methods and is suitable for evaluating difficult-controllable/-use floodwater in high-sediment rivers.

## Introduction

Flooding is an extreme hydrological phenomenon. Floods exhibit a double nature in that their occurrence represents both a natural disaster and an often vast quantity of water as a resource^1,2^. The occurrence and development of flooding is determined by both meteorological and geographical factors^3–5^. The complexity of flooding is evident in the fact that flood processes and water levels are affected by the geography of river basins and hydraulic factors in the flood zone^6^ in addition to human activities^7^ and global warming^8^. Flood disasters comprise a large portion of natural disasters worldwide^9,10^: Forty percent of global economic losses from natural disasters are caused by flooding^3^. In China, three main factors (land use practice, human settlements, and climate change) contribute to frequent flood disasters^1^, and ninety percent of large- and medium-sized cities are at risk of flooding to some extent^11^. Flooding causes more economic and property loss than any other types of disasters in China^11^.

In China’s Loess Plateau, the occurrence of floodwater is concentrated during the flood season (May–September). Moreover, the sediment concentration in run-off is extremely high, as much as 800 kg/m^3,^ ^12^. Loess Plateau run-off is believed to represent the highest sediment concentrations among the major rivers of the world, and run-off from the plateau contributes to the Yellow River^13–15^. China’s Loess Plateau faces significant water management challenges, resulting from water pollution and unique ecological challenges (including severe soil erosion). The high sediment concentration in run-off can significantly impact the utilisation of river run-off during flood season^16,17^. For example, the Jinghuiqu irrigation district in the Jing River downstream, located in the middle Loess Plateau, cannot make full use of the river water due to the hyper-concentration in run-off. The annual mean river water diversion from the river to the irrigation area during 1956–2001 was approximately 3.88 × 10^8^ m^3^, and only 32.2% occurred during the flood season, which caused not only severe water shortages in the irrigation district, but also floods downstream during flood season^12^. Thus, flood control and floodwater utilisation are attractive strategies for meeting the regional long-term water demand. As such, it is important to evaluate the availability of floodwater or floodwater that is difficult to control or use (*W*_*dcu*_) during flood season.

The concept of floodwater utilisation was proposed in 2005 by researchers and water managers possessing a deep understanding of the water resource shortages, water pollution, and ecological problems in China^18^, particularly in the Loess Plateau. Studies have focused on water resources management, reservoir flood control, and floodwater utilisation in the Loess Plateau and Yellow River Basin^12,19–21^. Li *et al*.^12^ evaluated the quantity of floodwater that is controllable but difficult to use (*W*_*c,du*_) with a new maximum grade approach (MGA) in the Jing River. Bai *et al*.^19^ presented a multi-objective optimal operation model of two pivotal reservoirs, Longyangxia and Liujiaxia, in the Upper Yellow River for evaluating reservoir gains and found that flood control in the downstream regions can be satisfied by the joint operation of the two reservoirs. Numerous similar studies have been performed globally, including the utilisation of rainwater and floodwater resources, floodwater control, the risks of floodwater, and flood management. Pinter^22^ discussed the effects of urbanisation and urban infrastructure on flood control in view of the reconstruction efforts in the USA after severe flooding in 1993. Chang *et al*.^23^ assessed the controllability of rain- and floodwater in Taipei, Taiwan. Ibrahim^24^ studied the utilisation of urban floodwater from precipitation in the arid region of central Sudan. Jonathan *et al*.^25^ studied the effects of dam construction and land use changes owing to flooding in the middle and lower reaches of the Mississippi River in the USA. Bower^26^ studied flood control in Canada’s Assiniboine River and concluded that flood disasters are closely related to man-made factors such as dykes and cut-offs. Srinivasan *et al*.^27^ proposed a concentrated–dispersed model for evaluating the rainwater utilisation policy in Chennai, India. Hsu *et al*.^28^ employed an adaptive network-based fuzzy inference system and a real-time recurrent learning neural network to develop a multi-phase intelligent real-time reservoir operation model for flood control in Taiwan. Uysal *et al*.^29^ developed a decision support system combining a hydrological (HEC-HMS) and reservoir simulation model (HEC-ResSim) to meet flood protection objectives in the Yuvacık Dam Basin, Turkey. Che and Mays^30^ simulated the real-time flood control of the Cumberland River at Nashville, Tennessee, USA, with an optimisation/simulation model including five major components. Hu *et al*.^31^ used a 1-D hydrodynamic model with real-time operations of sluices and pumps to quantitatively assess flood severity in Suzhou City, China. The aforementioned studies include flood-related research; however, studies that focus on *W*_*dcu*_ are not common. Generally, such specific research appears only in quantitative studies on the utility of surface water resources^12^. The availability of water at any time is largely governed by both the distribution of water and the hydrologic cycle of water in its various phases^32^. Theoretically, it is not difficult to calculate the quantity of usable river water^32–34^. However, owing to the number and complexity of influencing factors, calculating the actual quantity of usable water resources is difficult. There is still no reliable and effective way to assess the quantity of viable water resources, particularly for the Loess Plateau, where soil erosion is significant and the sediment content of rivers is high.

Currently, the quantity of *W*_*dcu*_ is primarily evaluated via two calculation methods^12^: the water consumption method and the floodwater rejection coefficient method. The former only considers the regulation capacity of engineering (i.e. reservoirs) and ignores the influence of water quality (i.e. sediment concentration in run-off) on floodwater utilisation^12^. Thus, it is not suitable for high-sediment rivers. Rivers can change from high to low sediment concentration and vice versa in different seasons, depending on the sediment delivered via run-off during those seasons (i.e. flood and dry seasons). For example, the Jing River in the middle Loess Plateau is a typical hyper-sediment river in flood season due to the high sediment concentration in run-off from severe soil erosion upstream, but it can become a low-sediment river in the low-flow season due to the low sediment concentration in run-off^16^. On the other hand, the floodwater rejection coefficient method only considers the ratio of annual maximum monthly flow to average monthly flow, but not the water quality, such as the sediment concentration in run-off^12^; it is therefore only applicable to low-sediment rivers. To address this shortcoming of the floodwater rejection coefficient method, Li *et al*.^12^ proposed a new approach, namely the MGA, to calculate the coefficient *β* in high-sediment rivers so as to further determine the quantity of *W*_*c,du*_ in high-sediment rivers. However, this method determines only the effect of sediment content of run-off on the quantity of *W*_*c,du*_ and neglects the effects of hydraulic engineering adjustments. Therefore, when evaluating the quantity of *W*_*dcu*_, it is difficult to directly apply existing traditional methods to high-sediment rivers, thus indicating the limitations of current methods in the case of high-sediment basins. Uncoordinated trends between run-off and sediment combinations appear to have important effects on the diversion and rejection of surface run-off in high-sediment basins during flood seasons^12^. The problem of evaluating the quantity of *W*_*dcu*_ in high-sediment rivers with flood control adjustment operations remains unresolved. Currently, no effective method is available to calculate the quantity of *W*_*dcu*_, and the factors influencing it remain unclear. Therefore, considering the previous studies, a method for evaluating the quantity of *W*_*dcu*_ in high-sediment rivers during the flood season is still needed.

The present study proposes a new method for evaluating the quantity of *W*_*dcu*_ from the combined perspectives of water quantity and quality in the Loess Plateau, with a case study of the Bajiazui Reservoir in the Jing River Basin. The new method has wide applicability for assessing W_*c,du*_ and evaluate the available surface water resources, thus providing a tool suitable for use by regional floodwater resources to solve water resource shortages in high-sediment rivers of Northwest China, especially those in the Loess Plateau.

## Results and Discussion

### Evaluation of the proposed method

**Figure 1:**
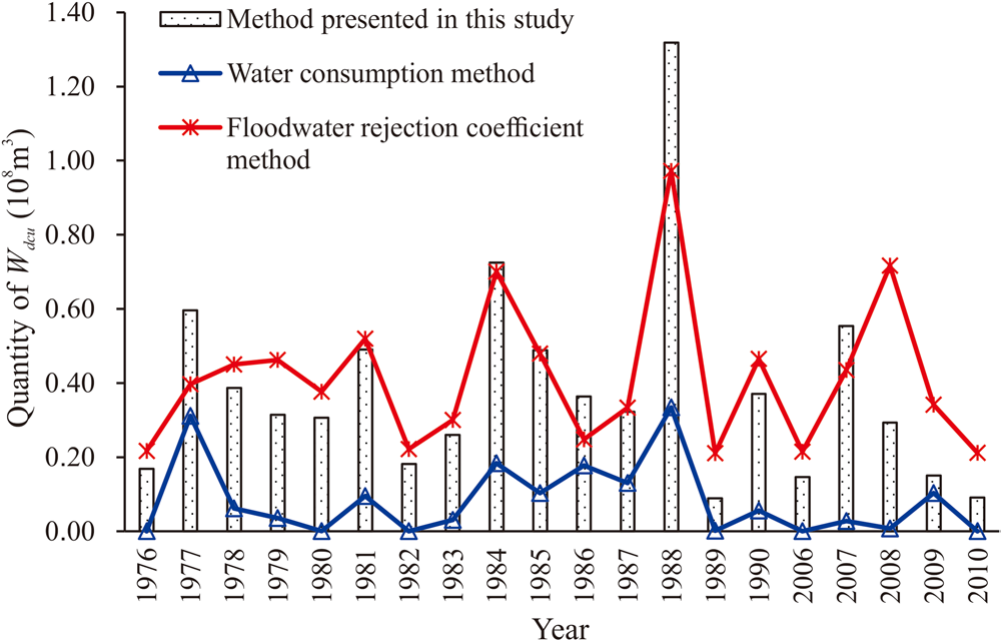
Comparison of the quantity of flood-season floodwater that is difficult to control or use (*W*_*dcu*_) between the two traditional methods (water consumption method and floodwater rejection coefficient method) and the method presented in this study at 10% of the sediment concentration limit of usable floodwater resource.

There are two traditional methods (water consumption method and floodwater rejection coefficient method) commonly used to evaluate the quantity of *W*_*dcu*_^12^. For the two traditional methods, the connection between water quantity and quality is not considered; that is, there is no distinction between *W*_*dcu*_, floodwater that is difficult to control (*W*_*dc*_), and *W*_*c,du*_. For the water consumption method, the quantity of *W*_*dcu*_ is only dependent on the regulation capacity of the Bajiazui Reservoir in this study, here equal to *W*_*dc*_ (equation (6)). For the floodwater rejection coefficient method, the quantity of *W*_*dcu*_ is calculated based on the rejection coefficient *β* in the range of 0.15–0.80, which relates to a ratio of annual maximum monthly flow to average monthly flow. When the value of this ratio is 1–2, the corresponding value of *β* is 0.15. When the ratio is 2–3, 3–4, 4–5, 5–6, 6–7, or greater than 7, the corresponding *β* values are 0.35, 0.45, 0.55, 0.85, 0.75, or 0.8, respectively. The results from these two traditional methods are shown in Fig. 1. The results of *W*_*dcu*_ from the floodwater rejection coefficient method are larger than those from the water consumption method and approach those from the method presented in this study. The results from the water consumption method are too small because it does not consider the quantity of *W*_*c,du*_ and are therefore not suitable to this case study.

**Figure 2:**
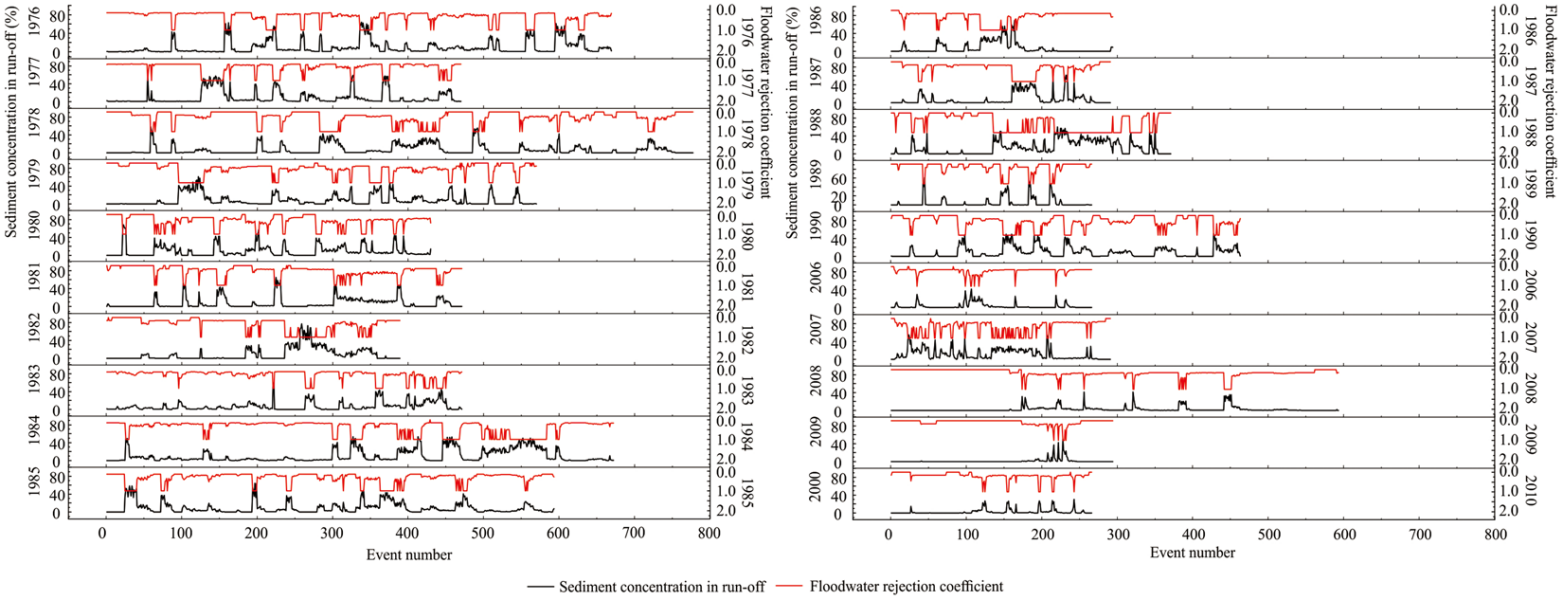
Calculated floodwater rejection coefficient (secondary y-axis with red solid line) based on the sediment concentration (y-axis with black solid line) of the reservoir’s release at 10% of the sediment concentration limit generated using OriginPro 9.1 for the Bajiazui Reservoir.

As shown in Fig. 2, the sediment concentration of the run-off exiting Bajiazui Reservoir is high. For example, between BT 02:00 on 15 July 1988 (event no. 136), and BT 12:00 on 11 September 1988 (event no. 352), the sediment content of the run-off was almost exclusively above 10%. The value of *β* calculated by using the traditional floodwater rejection coefficient method was always in the range of 0.15–0.80; therefore, a minimum of 15% of floodwater was *W*_*dcu*_ in every scenario. At the other extreme, a minimum of 20% of floodwater was considered as floodwater that is controllable and usable (*W*_*c,u*_), which is not consistent with the actual conditions. The relationship between the sediment concentration of run-off and river water utilisation/diversion is not considered. Thus, the traditional method, based on the ratios of annual maximum monthly run-off to monthly mean run-off, is only appropriate for use in rivers with a low run-off sediment concentration^12^. In contrast, during this period, the *β* value calculated by the MGA method was almost exclusively equal to 1 when the sediment concentration limit of usable floodwater resources was set as 10%, consistent with actual conditions. The MGA method used in this study considered the sediment concentration limit of the usable floodwater resources in the region. This new method considers the combined effects of water quantity and quality and divides *W*_*dcu*_ into *W*_*dc*_ and *W*_*c,du*_, calculated by using the flood control adjustment optimisation model and the MGA, respectively. The results are consistent with actual conditions; therefore, in high-sediment rivers, the method proposed in this paper is superior to traditional methods. This study has deepened our understanding of both the implications and calculation of *W*_*dcu*_.

The primary function of the Bajiazui Reservoir is flood prevention; other functions include water provision, irrigation, and electricity generation. Thus, adjustments made for flood prevention do not account for all adjustments made in reservoir operations even though the two are similar. However, the release from the reservoir and the run-off measured at the Bajiazui hydrological station downstream had correlation coefficients of 0.5 and above (Supplementary Fig. S1 **)**. All the correlation coefficients were above 0.50; therefore, the two quantities are significantly correlated with 99% confidence. This result demonstrates that the flood prevention adjustment model used in this study is accurate.

### Quantity of *W*_*dc*_

The duration of individual flood events varied owing to differences in the circumstances of each event in the Bajiazui Reservoir. To accurately model actual conditions, the periods used in the calculations had to match the actual durations of the individual events. I applied the improved genetic algorithm-simulated annealing (IGA-SA) hybrid algorithm proposed by Li and Wei^35^ to solve the flood control adjustment optimisation model by using real-value coding. The decision variable was the release from the reservoir; the population size was 100; the maximum number of iterations of the genetic algorithm was 100; the selection rate of the genetic algorithm was 0.25; the crossover rate was 0.75; and the mutation rate was 0.05. The initial temperature for simulated annealing was 1; the initial search neighbourhood was 0.30; the search space reduction factor was 0.97; the cooling rate was 0.94; the number of transitions at each temperature was 10; and the total number of temperature changes of simulated annealing was 90. When the termination conditions were satisfied, the individuals of release with the best fit were determined; the optimal value of each variable was the optimal release from the reservoir. The results are shown in Supplementary Fig. S2.

**Figure 3:**
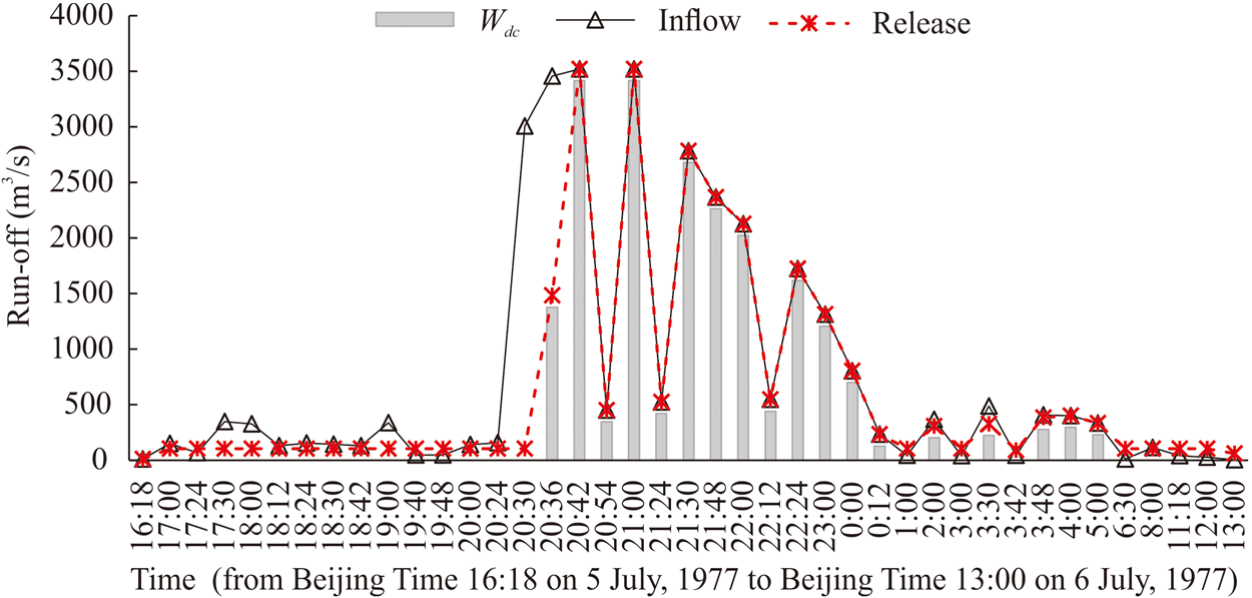
Quantity of floodwater that is difficult to control (*W*_*dc*_) resulting from the inflow events on 5 and 6 July 1977 for the Bajiazui Reservoir. The reservoir release was calculated based on the flood control adjustment optimisation model.

Supplementary Fig. S2 compares the inflow and release quantities following the flood adjustment optimisation at the reservoir. Most peak release values were smaller than the peak inflow values, owing to the reservoir flood control effects. During extremely large flood events, the inflow rate far exceeded the reservoir’s ability to adjust the water level in a controlled manner, and the excess water uncontrollably spilled from the reservoir. The floodwater released from the reservoir in this manner is *W*_*dc*_ (Fig. 3). Such uncontrollable spillage occurred during 16 of the 20 years included in this study (Supplementary Table S1). The average *W*_*dc*_ during each flood season was 10.4 million m^3^. The year with the largest amount of *W*_*dc*_ is 1988, with 33.4 million m^3^.

According to the PIII frequency analysis on the sequence of inflows at the Bajiazui Reservoir during 1962–2010 (see Supplementary Fig. S3), 8 years in Supplementary Table S1 showed high run-off, 10 years showed average run-off, and 2 years showed low run-off. The years with no *W*_*dc*_ were years with average (1976 and 1982) and low run-off (2006 and 2010). The annual peak inflow rates and annual quantity of *W*_*dc*_ were not strongly correlated. In 1982, the peak floodwater inflow rate was 473.6 m^3^/s with no *W*_*dc*_. In 1983, however, the peak floodwater inflow was only 428 m^3^/s, which was smaller than that in 1982, and *W*_*dc*_ was 3,106,000 m^3^.

### Values of *β* and *W*_*c,du*_

**Figure 4:**
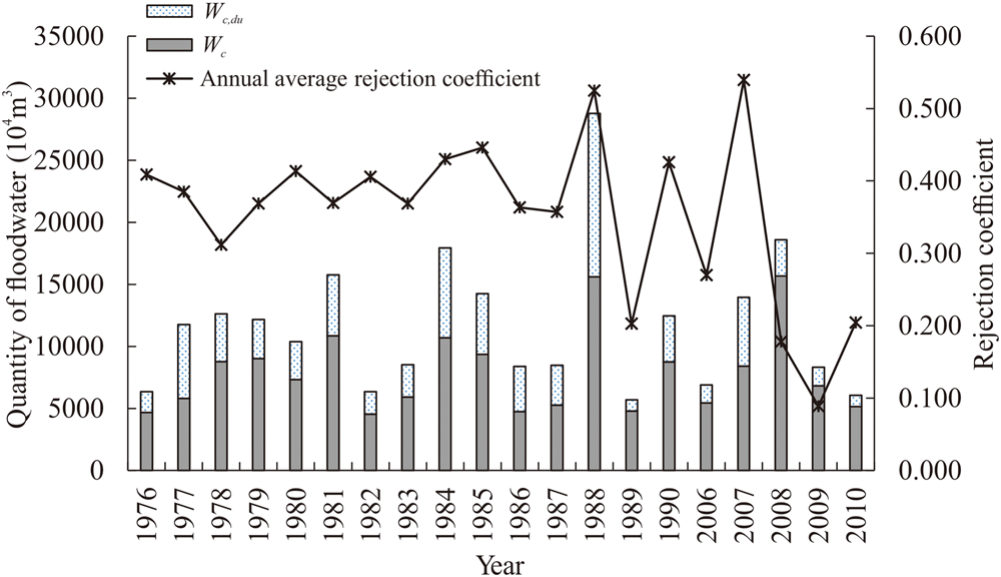
Calculated results of floodwater that is controllable (*W*_*c*_), floodwater that is controllable but difficult to use (*W*_*c,du*_), and annual average rejection coefficient using the new method presented in this study during 1976–1990 and 2006–2010 for the Bajiazui Reservoir. *W*_*c,du*_ and *W*_*c*_ are represented by the stacked histograms (y-axis), and the rejection coefficient is depicted with a solid line with stars (secondary y-axis).

The flood control adjustment optimisation model yields the reservoir’s release rate and the sediment concentration of run-off *C*_*q*_ (%). The values of *β* with a 10% sediment concentration limit of usable floodwater resource are shown in Fig. 4, according to equation (13). Higher sediment content in run-off leads to higher values of *β*. The square of their correlation coefficient was 0.9132. High quantities of controllable floodwater leaving the reservoir coupled with high *β* values led to high quantities of *W*_*c,du*_ (Fig. 4) based on equation (13). The highest quantity of controllable floodwater, 156.6 million m^3^, occurred during the 2008 flood season. However, the mean rejection coefficient of that year was low (0.178); therefore, the quantity of *W*_*c,du*_ was also low. During the 1988 flood season, the quantity of controllable floodwater was 156 million m^3^, which was slightly less than that during 2008. However, the mean rejection coefficient in 1988 was 0.525. The only year showing a higher mean rejection coefficient was 2007 (0.5390, although the quantity of *W*_*c*_ was significantly lower in that year. Therefore, the highest quantity of *W*_*c,du*_ (131.8 million m^3^) occurred in 1988.

These results describe a sediment concentration limit of 10%. A sediment limit set to a different value would change the mean rejection coefficient and quantity of *W*_*c,du*_, as shown in Supplementary Tables S2 and S3. These tables show that lower sediment limits lead to larger *β* values (Supplementary Table S2) and larger quantities of *W*_*c,du*_ (Supplementary Table S3). As the sediment limit increased, both *β* and *W*_*c,du*_ decreased.

### Quantity of *W*_*dcu*_

**Figure 5:**
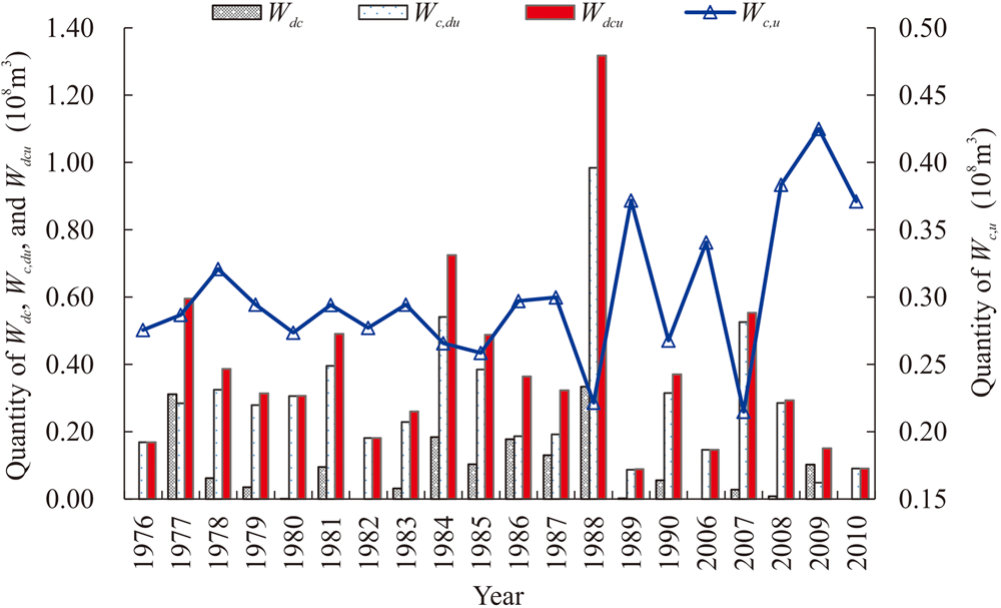
Relationships between floodwater that is difficult to control (*W*_*dc*_), floodwater that is controllable but difficult to use (*W*_*c*,*du*_), floodwater that is difficult to control or use (*W*_*dcu*_), and floodwater that is controllable and usable (*W*_*c,u*_) at a 10% sediment concentration limit for the Bajiazui Reservoir.

The quantity of *W*_*dcu*_ with a sediment concentration limit of 10% (Fig. 5) can be calculated using equation (1). On the right-hand side of the equation, we used the results of the flood control adjustment optimisation model (*W*_*dc*_) and the MGA (*W*_*c,du*_). Equation (15) can be used to determine the quantity of *W*_*c,u*_, which is also shown in Fig. 5 with a sediment concentration limit of 10%. The mean quantity of *W*_*dcu*_ was 38.1 million m^3^. Of that, 29.8 million m^3^ (78.21%) of the *W*_*dcu*_ was *W*_*c,du*_, and only 8.3 million m^3^ (21.79%) of the *W*_*dcu*_ was *W*_*dc*_. *W*_*c,du*_ was therefore the primary component of *W*_*dcu*_.

Table 1 depicts the mean annual quantities of *W*_*dcu*_ calculated by the proposed method and the traditional rejection coefficient method, which are 38.1 million m^3^ (at a 10% sediment concentration limit) and 41.4 million m^3^, respectively. These values represent 43.73% and 47.52% of the total release from the reservoir, respectively. The average quantity of *W*_*dcu*_ with a sediment concentration limit <8% calculated by the proposed method is slightly larger than that calculated by the traditional method.

**Table 1:** Comparison of the quantities of floodwater that is difficult to control or use (*W*_*dcu*_, 10^8^ m^3^) by using the proposed method versus the traditional rejection coefficient method at the Bajiazui Reservoir.

Year	Sediment concentration limit of usable floodwater resources (%)										Traditional method
	2	4	6	8	10	12	14	16	18	20	
1976	0.261	0.228	0.212	0.187	0.169	0.162	0.156	0.155	0.148	0.145	0.217
1977	0.705	0.639	0.627	0.606	0.596	0.589	0.578	0.566	0.561	0.558	0.397
1978	0.472	0.435	0.410	0.393	0.386	0.381	0.368	0.359	0.346	0.332	0.450
1979	0.522	0.414	0.378	0.346	0.314	0.293	0.291	0.277	0.273	0.270	0.462
1980	0.488	0.431	0.376	0.333	0.306	0.292	0.282	0.268	0.262	0.258	0.377
1981	0.630	0.552	0.516	0.498	0.491	0.478	0.428	0.425	0.421	0.417	0.520
1982	0.225	0.219	0.208	0.189	0.181	0.176	0.171	0.169	0.168	0.162	0.222
1983	0.393	0.342	0.309	0.286	0.260	0.252	0.248	0.246	0.238	0.226	0.300
1984	0.888	0.807	0.766	0.745	0.725	0.721	0.716	0.712	0.696	0.683	0.701
1985	0.694	0.579	0.532	0.507	0.488	0.461	0.438	0.424	0.419	0.398	0.479
1986	0.419	0.390	0.388	0.377	0.364	0.362	0.361	0.353	0.348	0.344	0.248
1987	0.387	0.371	0.341	0.325	0.323	0.320	0.312	0.309	0.308	0.308	0.333
1988	1.425	1.408	1.369	1.327	1.318	1.244	1.218	1.190	1.163	1.140	0.972
1989	0.107	0.102	0.101	0.094	0.089	0.089	0.081	0.075	0.075	0.072	0.211
1990	0.497	0.495	0.483	0.435	0.371	0.338	0.318	0.291	0.290	0.283	0.465
2006	0.192	0.173	0.158	0.150	0.146	0.139	0.130	0.127	0.116	0.112	0.215
2007	0.746	0.603	0.581	0.563	0.553	0.550	0.527	0.498	0.461	0.447	0.435
2008	0.533	0.362	0.314	0.298	0.293	0.290	0.284	0.274	0.272	0.267	0.717
2009	0.170	0.165	0.158	0.157	0.151	0.149	0.149	0.149	0.147	0.146	0.341
2010	0.205	0.116	0.106	0.093	0.091	0.091	0.089	0.088	0.088	0.085	0.212
Average	0.498	0.441	0.417	0.395	0.381	0.369	0.357	0.348	0.340	0.333	0.414

### Factors influencing *W*_*dcu*_

**Figure 6:**
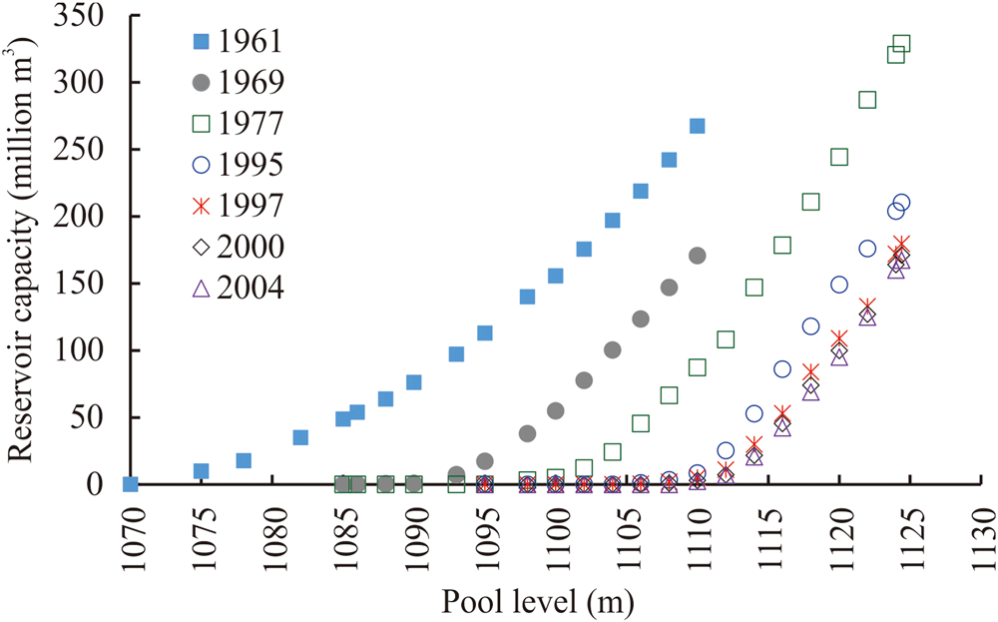
Pool level–capacity curves for the Bajiazui Reservoir in different years.

**Figure 7:**
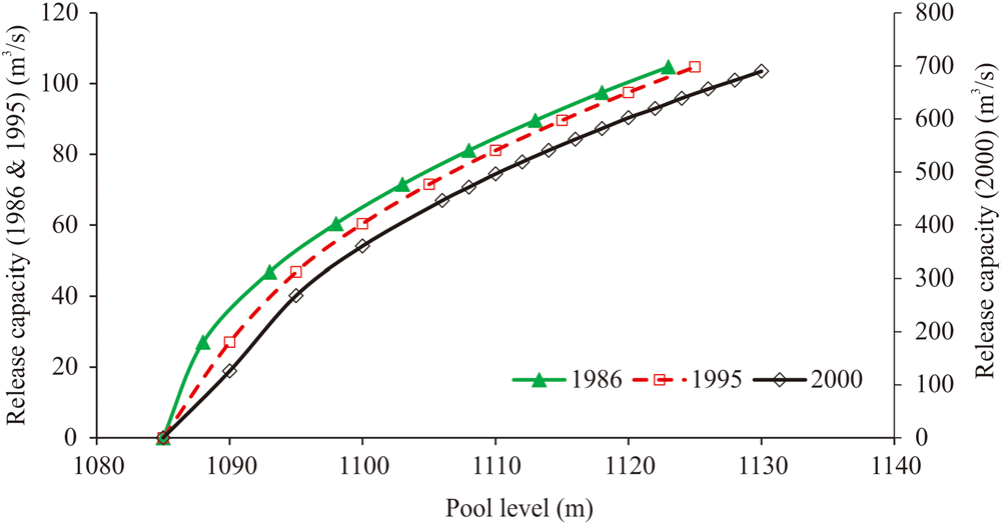
Pool level–release quantity curves for the Bajiazui Reservoir in 1986, 1995, and 2000.

The different factors influencing *W*_*dc*_ include reservoir capacity, release capacity, and flood control. These factors are shown in the pool level–reservoir capacity curve in Fig. 6, the pool level–release quantity curve in Fig. 7, and the reservoir’s flood control characteristic parameters in Table 2. Because the pool level–release rate relationship plotted in Fig. 7 begins at a water level of 1,085 m, this section is concerned with the pool level–reservoir capacity curve only above a pool level of 1,085 m in Fig. 6. The pool level–reservoir capacity curves from 1997, 2000, and 2004 are similar, although the 2004 curve was the outermost curve. Therefore, we considered a total of four pool level–reservoir capacity curves from 1969, 1977, 1995, and 2004, referred to as scenarios A, B, C, and D, respectively. For the pool level–release rate curves shown in Fig. 7, we considered only the curves from 1986 and 2000 (called scenarios I and II). Regarding the reservoir’s flood control characteristic parameters, we considered all eight sets of parameters given in Table 2 (called scenarios 1–8). Scenario 1 is the set of flood control characteristic parameters in the top row, from 1962 to 1974. Scenario 2 is the set of parameters in the second row from 1975 to 1978. Using different combinations of these scenarios, it was possible to construct 64 (i.e. 4 × 2 × 8) unique cases for analysis. We used the same IGA-SA method parameter settings as those described in the previous subsection to calculate the annual quantity of *W*_*dc*_. The 20-year average quantities of *W*_*dc*_ from each of the 64 scenarios are shown in Table 3.

**Table 2:** Characteristic parameters of flood prevention operation for the Bajiazui Reservoir.

Starting and ending years	Dead pool level (regulation start) (m)	Flood-control low-limit pool level (m)	Normal storage pool level (m)	Flood-control high-limit pool level (m)	Designed flood pool level (m)	Check flood pool level (m)	Height of dam (m)	Maximum floodwater release rate (m^3^/s)
1962–1974	1,085.0	1,100.0	1,102.0	1,103.7	1,108.4	1,108.4	1,108.7	104.7
1975–1978	1,085.0	1,110.0	1,112.0	1,115.0	1,119.9	1,124.4	1,124.7	104.7
1979–1980	1,086.0	1,110.0	1,112.0	1,115.0	1,119.9	1,124.4	1,124.7	104.7
1981	1,088.0	1,110.0	1,112.0	1,115.0	1,119.9	1,124.4	1,124.7	104.7
1982–1984	1,090.0	1,110.0	1,112.0	1,115.0	1,119.9	1,124.4	1,124.7	104.7
1985–1990	1,093.0	1,110.0	1,112.0	1,115.0	1,119.9	1,124.4	1,124.7	104.7
1991–1997	1,095.0	1,110.0	1,112.0	1,115.0	1,119.9	1,124.4	1,124.7	104.7
1998–present	1,095.0	1,109.0	1,112.0	1,115.0	1,119.9	1,124.4	1,124.7	689.8

**Table 3:** Mean quantity of floodwater that is difficult to control (*W*_*dc*_, 10^4^ m^3^) for different scenario combinations of the Bajiazui Reservoir in the Jing River during 1976–1990 and 2006–2010.

Pool level–reservoir capacity curve scenario (Fig. 6)	Pool level–release rate curve scenario (Fig. 7)	Reservoir flood control characteristic parameter scenarios (Table 2)							
		1	2	3	4	5	6	7	8
A	I	1,490.92	1,258.82	1,259.22	1,260.03	1,260.83	1,278.82	1,307.27	1,307.27
	II	371.40	271.37	271.45	271.59	271.74	275.00	280.33	280.33
B	I	1,794.98	962.51	962.55	962.60	962.65	962.72	962.77	962.77
	II	452.32	208.76	208.77	208.78	208.79	208.81	208.82	208.82
C	I	2,165.53	1,202.24	1,202.24	1,202.24	1,202.24	1,202.24	1,202.24	1,202.24
	II	576.63	252.19	252.19	252.19	252.19	252.19	252.19	252.19
D	I	2,211.53	1,329.68	1,329.68	1,329.68	1,329.68	1,329.68	1,329.68	1,329.68
	II	590.29	273.01	273.01	273.01	273.01	273.01	273.01	273.01

The different scenario combinations resulted in vastly different quantities of *W*_*dc*_. Among them, scenario I resulted in much higher quantities of *W*_*dc*_ than those in scenario II. The difference in the reservoir’s ability to regulate floodwater between scenarios I and II was highly significant. Among the reservoir’s flood control characteristic parameters, scenario 1 resulted in a greater amount of *W*_*dc*_ than scenarios 2–8 did, in which the quantity of *W*_*dc*_ either gradually increased, as in AI, AII, BI, and BII, or remained the same, as in CI, CII, DI, and DII.

The quantity of *W*_*dc*_ was determined mainly by the peak inflow of floodwater into the reservoir and the reservoir’s flood regulation capability. Figure 5 shows that for periods during which *W*_*dc*_ occurred, a high rate of floodwater, which exceeded the reservoir’s maximum flood control adjustment rate, flowed into the reservoir during or just prior to the same period. This indicates that the quantity of *W*_*dc*_ was related to several factors, including the peak floodwater inflow rate, the time distribution of the floodwater inflow, and the maximum adjustment rate of the reservoir. The quantity of *W*_*c,du*_, however, was closely related to the sediment content of the run-off and the usable sediment limit of water resources (Figs 2 and 4, and Supplementary Tables S2 and S3). Therefore, the elements that influenced the quantity of *W*_*dcu*_ were a complex combination of water quantity and quality, which demonstrates the complex relationships among hydrological, meteorological, social, and economic factors.

By combining the specific processes of calculating the quantity of *W*_*dc*_, this section quantitatively analyses the effects of three factors on the quantity of *W*_*dc*_: the size of the reservoir capacity for flood control (pool level–reservoir capacity curve, Fig. 6), the release capacity of the reservoir (pool level–release rate curve, Fig. 7), and the flood control characteristic parameters of the reservoir (Table 2). Table 3 presents the results of the 64 scenarios in terms of the quantity of *W*_*dc*_, which include all possible combinations of the four pool level–reservoir capacity curves (scenarios A–D), two pool level–release rate curves (scenarios I and II), and eight sets of reservoir flood control characteristic parameters (scenarios 1–8). Table 3 shows that, for a given water level–reservoir capacity curve, scenario I (the 1986 pool level–release rate curve) resulted in higher *W*_*dc*_ than scenario II (the 2000 pool level–release rate curve) did. This indicates that scenario II is superior to scenario I, which is attributable to the installation of release tunnels in 1997 that raised the reservoir’s maximum controllable release rate from 104.7 m^3^/s to 689.8 m^3^/s and thus greatly improved its flood control adjustability. For a given pool level–reservoir capacity curve and a given pool level–release rate curve, scenario 1 had the highest quantity of *W*_*dc*_. In rows AI, AII, BI, and BII of the table, scenario 2 has the smallest amount of *W*_*dc*_; as the scenarios progress from 3 to 8, the quantity of *W*_*dc*_ increases. In rows CI, CII, DI, and DII, the quantity of *W*_*dc*_ remains constant as the scenarios progress from 2 to 8. This indicates that scenarios 2–8 are superior to scenario 1, and scenarios 3–8 are not better than scenario 2. Table 2 shows that scenario 2 has the lowest dead pool level at which adjustment begins and the highest flood-control high-limit pool level. Therefore, scenario 2 has the greatest reservoir capacity for flood control. This is conducive to flood control adjustability. For a given pool level–release rate curve and a given set of flood control characteristic parameters, row B has the smallest quantity of *W*_*dc*_ in scenarios 2–8; row A has the lowest amount of *W*_*dc*_ in scenario 1. This indicates that scenario B is more optimal than scenarios A, C, and D. That is, the 1977 pool level–reservoir capacity curve is superior to those of 1969, 1995, and 2004. In Fig. 6, the pool level–reservoir capacity curves from 1969, 1977, 1995, and 2004 appear in order of left to right on the x-axis. Although the curve from 1969 is located the farthest to the left on the x-axis, the flood-control high-limit pool level in 1969 was only 1,103.7 m (Table 2). After the height of the dam was increased in 1975, the flood-control high-limit pool level rose to 1,115.0 m. Therefore, the 1977 pool level–reservoir capacity curve is superior to that of 1969; that is, scenario B is best. In summary, scenario combination BII2 is the ideal scenario because it resulted in the smallest quantity of *W*_*dc*_. Table 3 shows that, all things being equal, the progression from scenario I to scenario II resulted in the largest change in the quantity of *W*_*dc*_. Thus, the pool level–release rate curve was the main factor influencing the quantity of *W*_*dc*_. That is, the reservoir’s maximum release rate was the most important factor in optimizing flood control adjustment in the Bajiazui Reservoir.

After using the MGA process of calculating the quantity of *W*_*c,du*_, it was apparent that *β* was the primary factor influencing the quantity of *W*_*c,du*_. The value of *β*, in turn, was related to both the sediment concentration of the run-off and the sediment concentration limit of usable floodwater resources. High run-off sediment concentration led to high *β* values and high quantities of *W*_*c,du*_ (Figs 2 and 4, respectively). In contrast, low sediment-concentration limits for usable floodwater resources also led to high *β* values and high quantities of *W*_*c,du*_ (Supplementary Tables S2 and S3, respectively). According to the averages of *β* and *W*_*c,du*_ for each sediment concentration limit over 20 years in Supplementary Tables S2 and S3, the following linear equations were obtained relating the quantity of *W*_*c,du*_ (*y*, 10^8^ m^3^) to the sediment limit of usable floodwater (*x*_1_, %) and to *β* (*x*_2_): *y* = −0.0081* x*_1_ + 0.3940 (R² = 0.8927) and *y* = 0.7374* x*_2_ + 0.0352 (R² = 0.9984). The gradient of the function relating *y* and *x*_2_ (0.7374) is greater than the absolute value of the gradient of the function relating *y* and *x*_1_ (0.0081). This reveals that changing the value of *β* by a certain proportion has a greater effect on the quantity of *W*_*c,du*_ than changing the sediment limit of usable floodwater by the same proportion.

*W*_*dcu*_ is composed of two parts: *W*_*dc*_ and *W*_*c,du*_. This analysis revealed that the primary factor affecting *W*_*dc*_ was the release capacity of the reservoir and that affecting *W*_*c,du*_ was the value of *β* for a given quantity of *W*_*c*_. Under different sediment concentration limits of usable floodwater resources, *W*_*c,du*_ accounted for 75.07% (limit = 20%) to 83.35% (limit = 2%) of *W*_*dcu*_; only 16.65% (limit = 2%) to 24.93% (limit = 20%) was *W*_*dc*_. Therefore, the value of *β* was the most important factor influencing the quantity of *W*_*dcu*_. For other high-sediment rivers of the Loess Plateau or the Yellow River apart from the Jing River, similar conclusions should be drawn if the conditions of reservoir flood control characteristics and run-off-sediment generation relationships are similar to those of the Jing River, because they determine the two parts (*W*_*dc*_ and *W*_*c,du*_) of *W*_*dcu*_.

## Methods

### Overview

Located in the middle Loess Plateau of China (Supplementary Fig. S4), the Jing River is a typical high-sediment river^12^. The temporal-spatial distribution of water resources in the Jing River Basin is uneven. The water resource problem has become the primary limitation for socio-economic growth in the basin. The development of floodwater as a resource has significant applications in alleviating water shortages, increasing the efficiency of water resource usage, and mitigating flood disasters^18^. However, the utilisation of floodwater in the basin is difficult, because the sediment concentration of the run-off is extremely high during the flood season^12,16^. Therefore, it is important to evaluate *W*_*dcu*_.

Currently, the basin includes 126 reservoirs with a combined capacity of 793 million m^3^. The Bajiazui Reservoir, the only large reservoir in the basin, has a capacity of 511 million m^3^. This reservoir, completed and put into operation in 1961, is located in the middle reaches of the Pu River, a tributary of the Jing River. The dam controls a watershed of 3,472 km^2,36^.

Because the loess at the surface of the watershed controlled by the reservoir is loose, the run-off flowing into the reservoir after storms is often heavily laden with sediment. The average annual inflow is 130 million m^3^ of water, carrying 28,480,000 t of sediment. The flood season accounts for 53.85% and 79.78% of those amounts, respectively. Long-term trends show that water and sediment enter the reservoir mainly during July and August^36^. The current siltation conditions at the reservoir are critical, with sediment occupying 330 million m^3^ of the reservoir’s capacity.

### Data

The data used in this study were provided by the China Yellow River Water Conservancy Commission and included all floodwater inflow events into the Bajiazui Reservoir during 1976–1990 and 2006–2010, in addition to the measured run-off data from the Bajiazui Hydrological Station, located 500 m downstream of the dam. Furthermore, data of annual run-off that entered the Bajiazui Reservoir during 1962–2010 were collected. The inflow data indicated that all the large floodwater inflows occurred during the flood season from May to September. The Bajiazui Reservoir is adjusted for flood protection on a yearly basis (May–September).

Heavy siltation occurs in the Bajiazui Reservoir area owing to severe soil erosion in the watershed controlled by the reservoir. The pool level–capacity curve is dynamic (Fig. 6). The differences in the pool level–capacity curves for 1997, 2000, and 2004 were not large, which indicates that by 1997, the silt level in the reservoir had reached an equilibrium. This result is related to the construction of a flood release tunnel in 1997^36^. The reservoir’s flood control characteristic parameters are shown in Table 2.

### Concept of *W*_*dcu*_ and evaluation method

From the traditional perspective^37^, flood season floodwater can be divided into the portion that is controllable (*W*_*c*_) and that which is difficult to control (*W*_*dc*_); *W*_*dcu*_ is simply *W*_*dc*_. From this viewpoint, the traditional method ignores the relationship between water quantity and quality and makes no distinction between *W*_*dcu*_, *W*_*dc*_, and *W*_*c,du*_. However, in high-sediment rivers, floodwater resource utilisation is influenced by the engineering adjustment capacity and the sediment characteristics of run-off^16^. Therefore, calculations of *W*_*dcu*_ need to combine the regulating effects of reservoirs with the effects of floodwater quality on the utility of floodwater resources.

**Figure 8:**
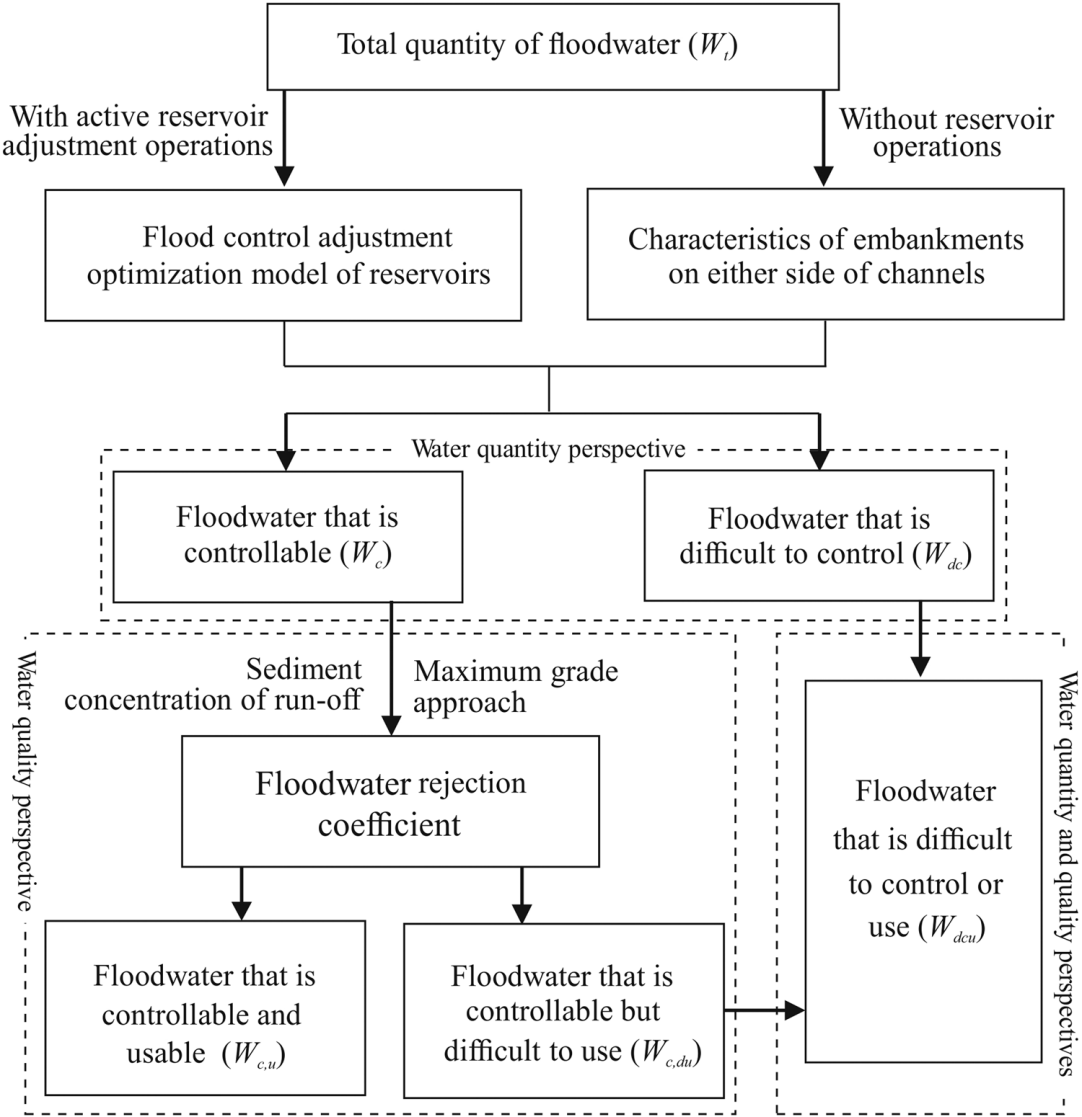
Schematic diagram of the relationships among the important elements of the quantitative and qualitative coupled evaluation of floodwater during the flood season (May–September).

From a water quality perspective, the total quantity of floodwater in the flood season (*W*_*t*_) can be divided into the quantity that is usable (*W*_*u*_ = *W*_*c,u*_) and that which is difficult to use (*W*_*du*_). From a quantity perspective, *W*_*t*_ can be divided into *W*_*c*_ and *W*_*dc*_. *W*_*c*_ can be further divided into *W*_*c,u*_ and *W*_*c,du*_ (Fig. 8). *W*_*dc*_ reflects operational adjustment capability, and *W*_*c,du*_ reflects quality requirements. Therefore, *W*_*dc*_ is fundamentally different from *W*_*c,du*_, because the two quantities have different natures; neither quantity alone completely reflects the components of *W*_*dcu*_.

Figure 8 shows that floodwater resources in the region are used in two ways: with active reservoirs and without hydraulic engineering operations. In both cases, *W*_*dc*_ is difficult to use. The portion of *W*_*c*_ with sediment content exceeding the sediment limit of usable run-off is *W*_*c,du*_. Run-off is composed of both *W*_*c*_ and *W*_*dc*_, although *W*_*c*_ may also contain *W*_*c,du*_. Thus, *W*_*dcu*_ includes the following two components:

1 $${W}_{dcu}={W}_{dc}+{W}_{c,du}$$

Thus we obtain the following:

2 $${W}_{c,u}={W}_{c}-{W}_{c,du}$$

or

3 $${W}_{c,u}={W}_{t}-{W}_{dcu}$$

This study examined a more complex case involving reservoir operations. In high-sediment rivers such as the Jing River, the sediment concentration of the run-off is the dominant factor affecting the utilisation of water resources^12^. Therefore, when the water quality was examined in this study, only the sediment concentration of the run-off was considered.

### Calculating *W*_*dcu*_

Step I: *W*_*dc*_ was determined based on the flood control adjustment optimisation model.

• Objective function

The objective of flood control operations at the Bajiazui Reservoir is to make full use of the reservoir’s storage and release capabilities to minimise losses during each flood event. Accordingly, the first objective is to minimise *W*_*dc*_:

4 $${\rm{\min }}({W}_{p1},{W}_{p2})$$

where *W*_*p*1_ is the adjusted release from the reservoir corresponding to a floodwater inflow of *p*_1_. Its maximum value is the maximum floodwater release rate in Table 2. *W*_*p*2_ is the maximum release from the reservoir that still prevents flooding downstream by meeting the downstream flood prevention flow standard of *p*_2_. The main considerations along the banks of the river downstream of the dam are 140,200 residents and 19,000 hm^2^ of farmland in Shaanxi and Gansu Provinces^36^. According to the embankment engineering grades and flood prevention standards given by Zhang and Wu^11^, this study used a 30–20 year return period as the standard for determining the designed flood for downstream flood control. Guo *et al*.^36^ reported that the corresponding peak floodwater flow rates at the Bajiazui Reservoir for designed floods with return periods of 30–20 years are 6,450 m^3^/s–5,270 m^3^/s. These values are significantly higher than those presented in Supplementary Table S1. That is, *W*_*p*2_ > *W*_*p*1_. Consequently, we obtained the rate of controlled discharge from the reservoir that satisfies flood control requirements *W*_*p*_ = min (*W*_*p*1_, *W*_*p*2_). Subsequently, we created the second objective function:

5 $${\rm{\min }}|{W}_{t0}-\,{\rm{\min }}\,({W}_{p},{W}_{tl})|$$

where *W*_*t*0_ is the release from the reservoir during time period *t*, and *W*_*tl*_ is the maximum controlled release from the reservoir during time period *t* corresponding to water level *l* in the reservoir. When *W*_*t*0_ > min (*W*_*p*_, *W*_*tl*_), the difference is *W*_*dc*_:

6 $${W}_{dc}={W}_{t0}-\,{\rm{\min }}\,({W}_{p},{W}_{tl})$$

Therefore, the quantity *W*_*c*_ is:

7 $${W}_{c}={W}_{t0}\mbox{--}{W}_{dc}$$

• Restrictions:

▯ Balance restriction of water quantity in the reservoir:

8 $${V}_{t+1}={V}_{t}+({I}_{t+1}+{I}_{t}){\rm{\Delta }}t/2-({O}_{t+1}+{O}_{t}){\rm{\Delta }}t/2$$

where *V*_*t*_ and *V*_*t*+1_ are the water volume in the reservoir at the beginning and end of time period *t*, respectively; *I*_*t*_ and *I*_*t*+1_ are the water flows into the reservoir at the beginning and end of time period *t*, respectively; *O*_*t*_ and *O*_*t*+1_ are the water flows from the reservoir at the beginning and end of time period *t*, respectively; and Δ*t* is the length of time period *t*.

▯ Release restriction:

9 $${O}_{t,{\rm{\min }}}\le {W}_{t0}\le {O}_{t,{\rm{\max }}}$$

where *O*_*t*,min_ and *O*_*t*,max_ are the minimum and maximum allowable releases during time period *t*, respectively. The minimum and maximum releases were set to 0 and min (*W*_*p*_, *W*_*tl*_), respectively.

▯ Reservoir capacity restriction:

10 $${V}_{t,{\rm{\min }}}\le {V}_{t}\le {V}_{t,{\rm{\max }}}$$

where *V*_*t*,min_ and *V*_*t*,max_ are the minimum and maximum allowable volumes of water stored in the reservoir during time period *t*, respectively. These values were set to the volumes of water in the reservoir corresponding to the levels of dead water and flood-control high-limit pool, respectively, and *V*_*t*_ is the volume of water stored in the reservoir during time period *t*.

▯ Non-negative restriction:

None of the variables involved can have negative values.

Step II: *W*_*c,du*_ was determined by the sediment concentration of the run-off. By analysing the relationship between the usage of floodwater in the basin and the sediment concentration of the run-off, the relationships between *β* and the sediment concentration of run-off (*C*_*q*_) could be determined as follows:

11 $$\beta =\{\begin{array}{l}\begin{array}{ccc}0 & {\rm{if}} & {C}_{q}=0\end{array}\\ \begin{array}{ccc}1 & {\rm{if}} & {C}_{q}\ge {C}_{q1}\end{array}\\ \begin{array}{cc}f({C}_{q}) & {\rm{others}}\end{array}\end{array}$$

where *C*_*q*1_ is the sediment concentration limit of the usable floodwater.

In equation (11), *f*(*C*_*q*_) is a function that relates *β* and *C*_*q*_ and is determined by using the MGA proposed by Li *et al*.^12^. This method was used to quantitatively deduce *β* in high-sediment rivers and is based on the characteristics of water and sediment sources in the channel and the effects of sediment concentration on the usability of water resources^12^. The monthly data series, including the quantity of run-off, the sediment content of run-off, and the quantity of water diverted during 1933–2010, was used to derive the following formula:

12 $$f({C}_{q})=0.0197\,{C}_{q}+0.1505\,({{\rm{R}}}^{2}=0.9754)$$

Thus *W*_*c,du*_ could be determined as follows:

13 $${W}_{c,du}=\beta {W}_{c}$$

Step III: According to *W*_*dc*_ from Step I and *W*_*c,du*_ from Step II, *W*_*dcu*_ could be determined in terms of equation (1).Then, *W*_*c,u*_ could be obtained as follows:

14 $${W}_{c,u}={W}_{t0}-{W}_{dcu}$$

or

15 $${W}_{c,u}=({W}_{t0}-{W}_{dc})(1-\beta )={W}_{c}(1-\beta )$$

### Data availability

The datasets generated during and/or analysed during this study are available at the Yellow River Conservancy Commission of the Chinese Ministry of Water Resources (http://www.yellowriver.gov.cn/).

## Electronic supplementary material

Supplementary Information
