# Supplementary material for: A new method to evaluate floodwater for control/use in high-sediment rivers of Northwest China
Source: Sci Rep. 2017 Dec 8;7:17219. doi: 10.1038/s41598-017-17489-6 (PMC5722886; doi:10.1038/s41598-017-17489-6)
Supplement: Supplementary file 1 — Supplementary Information [file 41598_2017_17489_MOESM1_ESM.pdf]

Supplementary information

A new method to evaluate floodwater for control/use in high-sediment rivers of Northwest China

Xungui Li[1,2,*]

[1]Key Laboratory of Western China's Environmental Systems (Ministry of Education), College of Earth and Environmental Sciences, Lanzhou University, 222 South Tianshui Road, Lanzhou, Gansu Province 730000, China
[2]Research Center for Arid Region and Desert, Lanzhou University, 222 South Tianshui Road, Lanzhou, Gansu Province 730000, China
*lixungui@163.com, lixung@lzu.edu.cn

Supplementary Table S1. Run-off category, occurrence time of the highest peak inflow rate, and the quantity of floodwater that is difficult to control (W_{dc}) for the annual average inflow into the Bajiazui Reservoir

Year	Annual average inflow (10^4 m^3)	PIII frequency P (%)	Run-off category*	Highest peak inflow rate (m^3/s)	Occurrence time and date of highest peak inflow rate	W_{dc} (10^4 $m^{3)}$
1976	9,858	41.16	AY	193.7	BT 16:48 on 15 Sep.	0
1977	12,700	24.85	HRY	3,520	BT 20:42 on 5 Jul.	3,112.9
1978	10,200	38.79	AY	1,003.2	BT 00:12 on 21 Jul.	615.2
1979	8,460	52.08	AY	1,009.6	BT 22:48 on 28 Jul.	352.6
1980	10,500	36.88	HRY	452.8	BT 10:18 on 18 Aug.	5.2
1981	11,700	29.79	HRY	1,098	BT 01:30 on 15 Aug.	950.6
1982	9,540	43.59	AY	473.6	BT 19:18 on 10 Aug.	0
1983	8,860	48.77	AY	428	BT 04:18 on 7 Sep.	310.6
1984	14,000	19.70	HRY	1,482.9	BT 17:30 on 24 Aug.	1,838.9
1985	11,200	32.53	HRY	1,728	BT 23:24 on 1 May	1,029.2
1986	8,110	55.14	AY	2,483.4	BT 9:00 on 26 Jun.	1,777.0
1987	8,540	51.39	AY	2,336	BT 1:18 on 28 Jul.	1,303.5
1988	16,000	14.23	HRY	1,595.2	BT 5:00 on 23 Jul.	3,339.9
1989	7,595	59.64	AY	345.6	BT 2:42 on 10 Aug.	17.78
1990	11,238	32.32	HRY	801.5	BT 23:12 on 25 Aug.	553.6
2006	7,091	64.74	LRY	356.9	BT 8:00 on 4 Aug.	0
2007	9,331	45.18	AY	4,172.5	BT 2:13 on 28 Jul.	275.0
2008	10,582	36.35	HRY	1,985.5	BT 0:00 on 2 May	78.2
2009	7,951	56.53	AY	3,951.8	BT 9:12 on 26 Jun.	1,025.6
2010	6,394	71.93	LRY	508.4	BT 3:00 on 10 Aug.	0

*AY: average year; HRY: high run-off year; LRY: low run-off year. According to China's national standard for terms and symbols used in hydrology, GB/T50095-98, the annual run-off frequency P, calculated by using the PIII frequency curve, divides run-off into five categories: very high run-off years ($P \leq 12.5\%$), high run-off years ($12.5\% < P \leq 37.5\%$), average run-off years ($37.5\% < P \leq 62.5\%$), low run-off years ($62.5\% < P \leq 87.5\%$), and very low run-off years ($P > 87.5\%$).

Supplementary Table S2. Floodwater rejection coefficients under different sediment concentration limits of usable floodwater resources

Year	Sediment concentration limit of usable floodwater resources (%)									
	2	4	6	8	10	12	14	16	18	20
1976	0.655	0.560	0.501	0.445	0.409	0.387	0.375	0.367	0.354	0.349
1977	0.584	0.473	0.438	0.414	0.385	0.370	0.366	0.352	0.343	0.340
1978	0.463	0.416	0.366	0.330	0.312	0.302	0.289	0.277	0.268	0.258
1979	0.584	0.487	0.445	0.406	0.369	0.346	0.338	0.322	0.314	0.311
1980	0.660	0.583	0.502	0.454	0.413	0.382	0.367	0.345	0.331	0.320
1981	0.475	0.424	0.403	0.388	0.369	0.341	0.301	0.294	0.283	0.271
1982	0.487	0.479	0.466	0.431	0.406	0.391	0.371	0.364	0.358	0.342
1983	0.652	0.537	0.472	0.404	0.369	0.346	0.333	0.325	0.314	0.309
1984	0.664	0.534	0.474	0.453	0.430	0.424	0.419	0.416	0.406	0.395
1985	0.704	0.580	0.513	0.468	0.446	0.414	0.382	0.365	0.356	0.345
1986	0.474	0.425	0.415	0.395	0.363	0.357	0.351	0.341	0.325	0.321
1987	0.473	0.437	0.393	0.364	0.357	0.346	0.327	0.320	0.313	0.309
1988	0.621	0.608	0.572	0.543	0.525	0.497	0.480	0.462	0.451	0.440
1989	0.233	0.221	0.219	0.205	0.203	0.203	0.189	0.177	0.173	0.164
1990	0.542	0.537	0.509	0.466	0.426	0.394	0.369	0.336	0.328	0.315
2006	0.355	0.322	0.288	0.275	0.270	0.260	0.247	0.232	0.222	0.215
2007	0.825	0.639	0.590	0.558	0.539	0.528	0.506	0.483	0.456	0.438
2008	0.329	0.245	0.200	0.186	0.178	0.177	0.171	0.167	0.165	0.164
2009	0.126	0.113	0.100	0.098	0.089	0.087	0.087	0.087	0.083	0.082
2010	0.352	0.268	0.231	0.207	0.204	0.200	0.197	0.193	0.193	0.188

Supplementary Table S3. Quantities of floodwater that is controllable but difficult to use ($W_{c,du}$, 10^8 m^3) under different sediment concentration limits of usable floodwater resources

Year	Sediment concentration limit of usable floodwater resources (%)									
	2	4	6	8	10	12	14	16	18	20
1976	0.261	0.228	0.212	0.187	0.169	0.162	0.156	0.155	0.148	0.145
1977	0.393	0.328	0.316	0.295	0.596	0.278	0.267	0.254	0.250	0.247
1978	0.410	0.374	0.348	0.331	0.386	0.319	0.306	0.297	0.285	0.271
1979	0.487	0.378	0.343	0.311	0.314	0.258	0.256	0.241	0.237	0.235
1980	0.488	0.431	0.375	0.333	0.306	0.292	0.281	0.268	0.261	0.257
1981	0.535	0.457	0.421	0.403	0.491	0.383	0.333	0.330	0.326	0.322
1982	0.225	0.219	0.208	0.189	0.181	0.176	0.171	0.169	0.168	0.162
1983	0.362	0.311	0.278	0.255	0.260	0.221	0.217	0.215	0.207	0.194
1984	0.704	0.623	0.582	0.561	0.725	0.537	0.533	0.528	0.513	0.499
1985	0.592	0.476	0.429	0.404	0.488	0.358	0.335	0.322	0.316	0.296
1986	0.241	0.212	0.210	0.199	0.364	0.184	0.183	0.176	0.170	0.166
1987	0.257	0.240	0.211	0.195	0.323	0.189	0.182	0.179	0.178	0.178
1988	1.091	1.074	1.035	0.993	1.318	0.910	0.884	0.856	0.829	0.806
1989	0.105	0.100	0.100	0.092	0.089	0.087	0.079	0.074	0.074	0.070
1990	0.442	0.439	0.427	0.379	0.371	0.282	0.262	0.236	0.234	0.227
2006	0.192	0.173	0.158	0.150	0.146	0.139	0.130	0.127	0.116	0.112
2007	0.718	0.576	0.554	0.536	0.553	0.523	0.500	0.471	0.433	0.419
2008	0.526	0.354	0.306	0.290	0.293	0.282	0.276	0.267	0.264	0.259
2009	0.068	0.062	0.055	0.054	0.151	0.047	0.047	0.047	0.044	0.044
2010	0.205	0.116	0.106	0.093	0.091	0.091	0.089	0.088	0.088	0.085

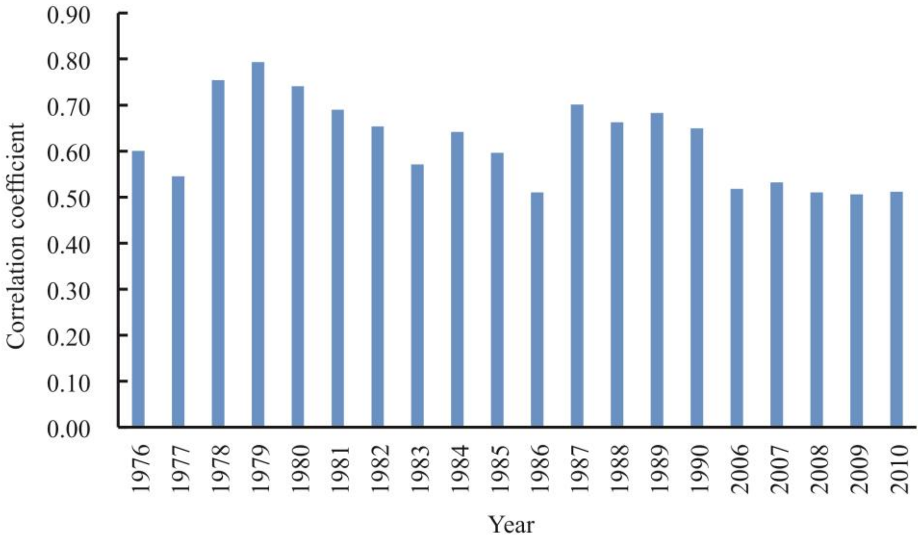

Supplementary Figure S1. Correlation coefficients between the optimised release of floodwater from the Bajiazui Reservoir for flood prevention and the actual run-off measured at the Bajiazui hydrological station downstream of the reservoir.

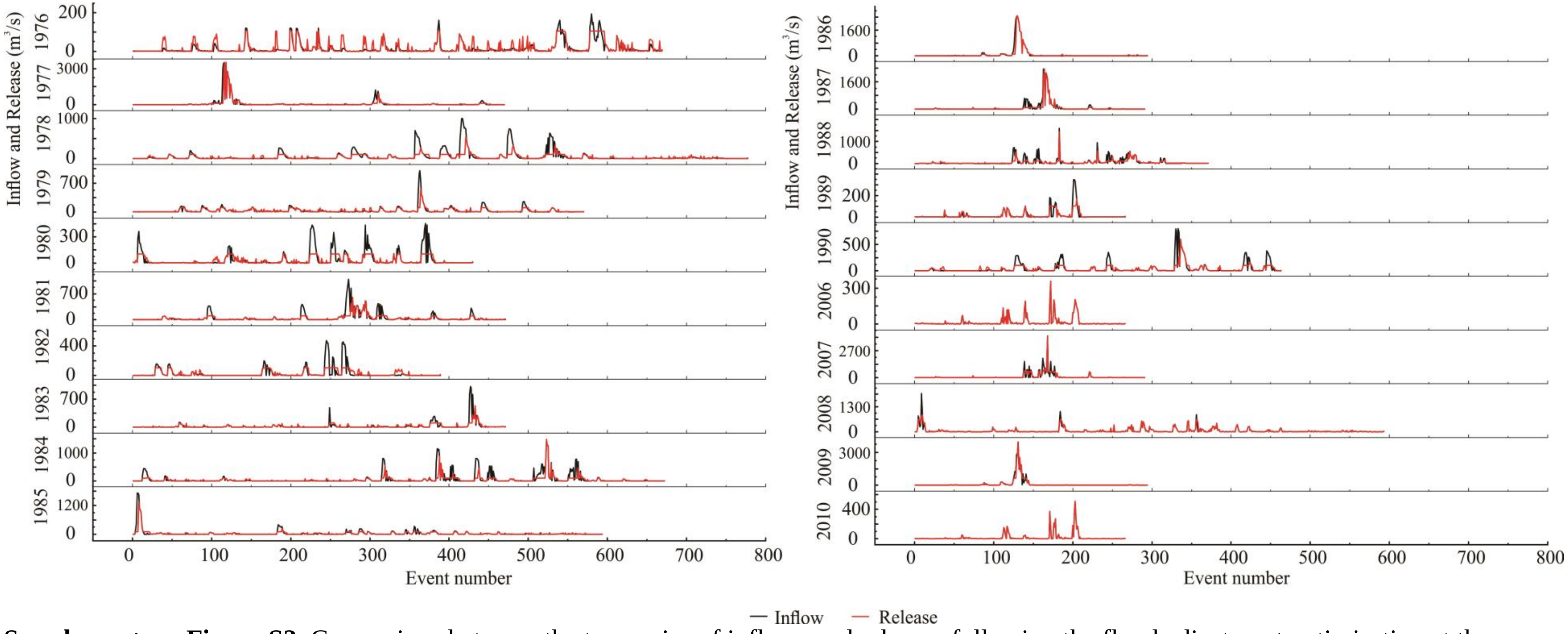

Supplementary Figure S2. Comparison between the two series of inflows and releases following the flood adjustment optimisation at the Bajiazui Reservoir generated using OriginPro 9.1.

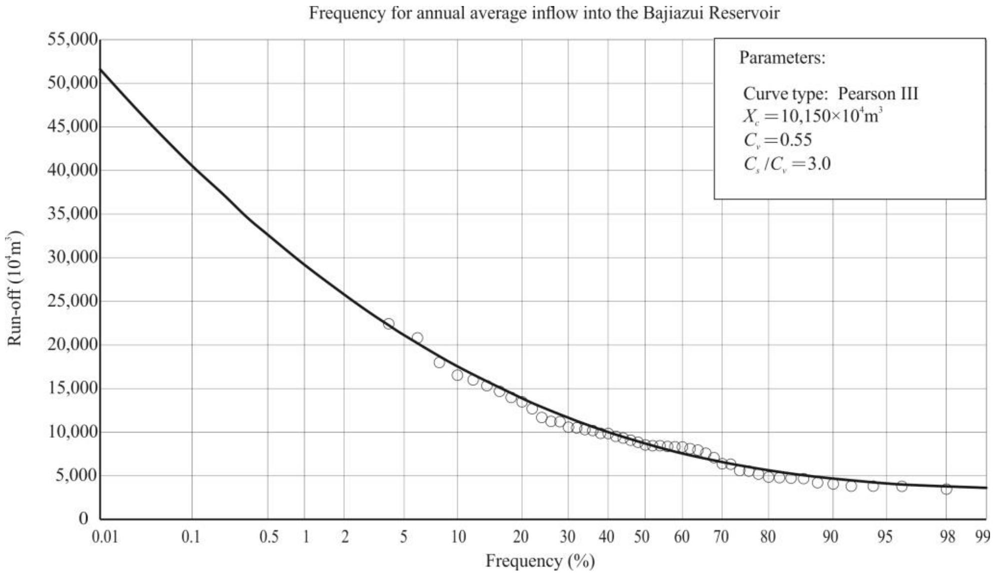

Supplementary Figure S3. Results of PIII frequency analysis on the data series of annual average inflows into the Bajiazui Reservoir during 1962–2010. X_c is the average annual run-off; C_v is the coefficient of variation; C_s is the coefficient of skewness.

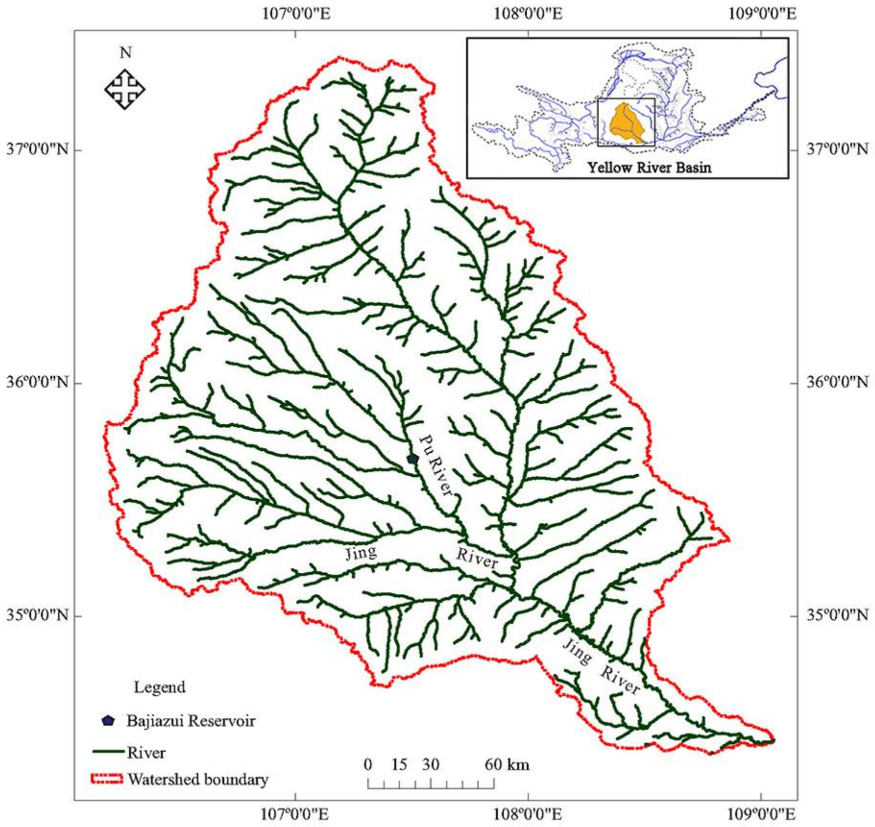

Supplementary Figure S4. Locations of the Jing River Basin and the Bajiazui Reservoir. Map of the Jing River Basin was generated using ArcMap 10.0 (http://appsforms.esri.com/products/download/index.cfm?fuseaction= download.all#ArcGIS_Desktop) based on a 30 m resolution digital elevation model (https://geography.wr.usgs.gov/sfcreek/dem.html). The inset of the Yellow River Basin was created using Adobe Photoshop CS6 (http://www.adobe.com/cn/products/cs6/photoshop.html). The two maps were combined using Adobe Photoshop CS6.
